# Augmented Reality Integration in Surgery for Craniosynostoses: Advancing Precision in the Management of Craniofacial Deformities

**DOI:** 10.3390/jcm14124359

**Published:** 2025-06-19

**Authors:** Divya Sharma, Adam Matthew Holden, Soudeh Nezamivand-Chegini

**Affiliations:** 1 University Hospitals Bristol & Weston, Bristol, BS2 8HW, UK; 2 Department of Oral & Maxillofacial Surgery, Honorary Clinical Lecturer for University College London, London WC1E 6B5, UK

**Keywords:** augmented reality, craniofacial deformities, craniosynostosis, navigation

## Abstract

Craniofacial deformities, particularly craniosynostosis, present significant surgical challenges due to complex anatomy and the need for individualised, high-precision interventions. Augmented reality (AR) has emerged as a promising tool in craniofacial surgery, offering enhanced spatial visualisation, real-time anatomical referencing, and improved surgical accuracy. This review explores the current and emerging applications of AR in preoperative planning, intraoperative navigation, and surgical education within paediatric craniofacial surgery. Through a literature review of peer-reviewed studies, we examine how AR platforms, such as the VOSTARS system and Microsoft HoloLens, facilitate virtual simulations, precise osteotomies, and collaborative remote guidance. Despite demonstrated benefits in feasibility and accuracy, widespread clinical adoption is limited by technical, ergonomic, financial, and training-related challenges. Future directions include the integration of artificial intelligence, haptic feedback, and robotic assistance to further augment surgical precision and training efficacy. AR holds transformative potential for improving outcomes and efficiency in craniofacial deformity correction, warranting continued research and clinical validation.

## 1. Introduction

Craniofacial deformities present significant surgical challenges due to the complex hard and soft tissue anatomical variability, functional considerations, and aesthetic outcomes. Craniosynostosis is the premature fusion of one or more cranial sutures in infants, which can cause skull deformity, elevated intracranial pressure, and, in certain cases, impaired brain development. Surgical correction typically involves cranial vault remodeling or endoscopic suture release, often done early in life. Because each case is unique, detailed preoperative planning is essential.

Traditional approaches rely on preoperative 2D/3D imaging, surgical guides, and navigation systems. However, limitations in spatial awareness and real-time anatomical referencing can impede intraoperative precision. Superimposing digital anatomical data with augmented reality (AR) onto the physical surgical field enhances situational awareness and may mitigate these limitations. As AR technologies continue to evolve, they offer promising tools for improving surgical accuracy, reducing operative time, and enhancing postoperative outcomes in craniofacial procedures. This paper aims to explore the applications of AR in pre-operative planning and simulation, intra-operative navigation, and education and telemedicine with regards to craniofacial deformity surgery.

## 2. Materials and Methods

We conducted a narrative literature review using PubMed and Google Scholar with search terms including “augmented reality,” “craniofacial deformities,” “craniosynostosis,” and “navigation.” Our inclusion criteria were peer-reviewed papers in English centering on augmented reality applications for preoperative planning, intraoperative navigation, and education and training in craniosynostosis surgery. Our exclusion criteria were non-English language papers. Papers published within the last 10 years were included.

## 3. Clinical Applications in Craniofacial Deformity Surgery

### 3.1. Preoperative Planning and Simulation

Craniosynostosis surgery demands meticulous preoperative planning due to the complex variable cranial anatomy and its direct implications for neurodevelopment, cranial aesthetics, and intracranial pressure regulation. AR has emerged as a transformative tool in this domain, enhancing surgical planning and simulation with unprecedented precision.

AR facilitates the intraoperative visualisation of deep anatomical structures by superimposing virtual models onto the physical surgical field in real time. This is achieved through the integration of patient-specific three-dimensional reconstructions, derived from preoperative computed tomography (CT) or magnetic resonance imaging (MRI) data, which are projected onto the patient’s anatomy via AR head-mounted displays or tablet-based systems [1]. Such technology enables surgeons to interact with a virtual representation of the cranial anatomy, allowing comprehensive assessment of suture fusion and cranial deformities from multiple perspectives. This enhanced spatial awareness significantly aids in the evaluation of synostosis morphology and severity. Preliminary studies [2,3] indicate that the incorporation of AR into preoperative planning and intraoperative guidance may improve the safety, accuracy, and efficacy of craniofacial surgical interventions by reducing intraoperative uncertainty and optimising decision-making.

Using AR platforms such as the VOSTARS (Video and Optical See-Through Augmented Reality Surgical System), surgeons can simulate osteotomies and cranial remodelling procedures on virtual patient-specific models with sub-millimetric accuracy (±1.0 mm) [4]. These simulations allow for the preoperative evaluation of various surgical strategies, prediction of postoperative cranial morphology and intracranial volume, and iterative adjustments to enhance symmetry, all of which contribute to reduced operative time and improved outcomes.

Some systems allow manipulation of virtual bones to plan repositioning, reshaping, and reconstruction. The capability of certain systems to allow manipulation of virtual bones for planning is well-documented in the literature on virtual surgical planning (VSP) and AR applications [5].

More recently, Maria et al. [3] discussed the adaptation of virtual surgical planning to midface reconstruction. This study evaluated the use of Microsoft HoloLens 2, an AR headset, as a tool for intraoperative surgical navigation in paediatric craniofacial surgery. Following initial validation in a preclinical setting using 36 simulated procedures on a 3D-printed patient model, the system was subsequently tested in vivo on 10 paediatric patients.

For each clinical case, virtual surgical planning was performed and integrated into both the AR interface and a standard neurosurgical navigation system. Surgeons delineated osteotomy lines using both modalities, and accuracy was assessed using calibrated CAD/CAM cutting guides. In preclinical trials, HoloLens 2 demonstrated an accuracy within ±1.5 mm. In clinical application, osteotomies performed under AR guidance met the ±1.5 mm accuracy threshold in 45% of the traced line (±0.4 SD) and 34% for a stricter ±1 mm threshold. By comparison, the standard neurosurgical navigator achieved 36% and 16%, respectively, at the same thresholds [6].

Additionally, AR systems facilitate collaborative planning by allowing surgeons, radiologists, and prosthetic technicians to engage with a shared virtual model. This real-time interaction enables the exchange of insights and the refinement of strategies, promoting an interdisciplinary approach that enhances surgical outcomes and ensures a unified treatment plan for the patient [7].

These studies underscore the utility of AR and VSP technologies in enabling clinicians to interact with and modify virtual representations of patient anatomy, thereby enhancing preoperative planning and potentially improving surgical outcomes.

### 3.2. Intraoperative Navigation

Application of AR to guide surgery has been studied across a multitude of disciplines [6]. The most transformative impact of AR in maxillofacial surgery is its capacity to deliver real-time, intraoperative guidance. Traditionally, surgeons have depended on static cutting guides or navigation systems that necessitate looking away from the surgical site to consult external monitors. AR overcomes this challenge by overlaying essential information directly onto the patient’s anatomy, enabling surgeons to keep their attention on the operative field throughout the procedure.

AR proves beneficial in procedures that involve harvesting and placing bone grafts. For example, in fibula free flap surgeries, AR systems project virtual templates of bone segments, enabling precise cuts and accurate alignment during reconstruction [8]. This reduces the reliance on pre-made cutting guides, which lack flexibility in adapting to intraoperative changes, such as unforeseen tumour growth.

In addition, intraoperative AR integrates effectively with robotic surgical systems. Surgeons can overlay AR visuals to improve the accuracy of robotic tools, combining the advantages of automation with real-time visual guidance [9]. This hybrid technique has shown great potential in invasive maxillofacial surgeries, where even small errors can have significant consequences [10].

AR has been effectively applied in the surgical management of synostotic plagiocephaly, offering real-time navigational guidance through positional and orientation data during open calvarial reconstruction [11]. In this study, seven patients (aged 6–24 months) underwent AR-assisted surgery. Preoperative planning was conducted using three-dimensional computed tomography (3D CT) data, and image registration via predefined anatomical markers enabled the intraoperative overlay of virtual anatomical structures, facilitating accurate osteotomies. The AR system was successfully implemented in all cases, providing consistent intraoperative support. Postoperative assessments revealed a significant reduction in cranial asymmetry, with intracranial volume asymmetry decreasing from 27.87% to 16.57%.

In a phantom study, Thabit et al. [12] evaluated the Microsoft HoloLens-based AR-electromagnetic (AR-EM) tracking system for its utility in delineating cranial sutures and guiding spring-assisted craniectomies. The system achieved a mean delineation error of approximately 2.4 ± 1.2 mm, within the clinically acceptable threshold of 5 mm, demonstrating both precision and adaptability across anatomical variations. The coronal suture exhibited the highest accuracy (~2 mm), while the lambdoid suture showed greater error (~3.3 mm), likely due to its distance from the anteriorly placed registration landmarks. The authors concluded that the system’s accuracy is likely replicable in clinical settings, contingent upon patient alignment and stable tracking, with future clinical validation planned.

Similarly, Garcia-Mato et al. [13] investigated the use of AR in five cases of open cranial vault remodelling for craniosynostosis. A real-time tracking system based on optical markers was developed to guide surgeons in placing bone fragments. This system achieved sub-millimetric accuracy without extending operating time and did not require invasive fixation or rigid immobilisation of the patient. Their study concluded that integrating technologies such as CranioPlan, 3D photography, navigation, and AR can significantly enhance surgical precision, consistency, and educational training in craniosynostosis treatment. AR has additionally been applied in posterior distraction for craniosynostosis. Through utilising virtual visualisation of the transverse sinus to guide the posterior cranial vault osteotomy, osteotomy lines were designed safely and efficiently [14].

AR-guided osteotomies have shown improved alignment with preoperative surgical plans. In one clinical study, 45% of osteotomy lines traced using AR met a ±1.5 mm accuracy threshold, compared to only 36% when using conventional neuronavigational techniques [15]. This highlights AR’s potential to enhance surgical precision in craniofacial procedures.

Studies have reported up to a 20% reduction in operative time during craniosynostosis repair when using augmented reality systems. This improvement is primarily attributed to more streamlined intraoperative workflows, where real-time anatomical overlays reduce the need for repeated image referencing and manual measurements, thereby enhancing surgical efficiency [16].

The use of augmented reality in cranial reconstruction has been associated with improved symmetry in postoperative outcomes. In one clinical series, volume asymmetry was reduced from 27.9% preoperatively to 16.6% following surgery, indicating a significant enhancement in cranial shape consistency with AR-guided techniques [11].

### 3.3. Education and Telemedicine

AR also holds significant promise in surgical education and training. Hybrid AR simulation platforms have been developed to enhance preoperative planning and skill acquisition in craniofacial surgery. Coelho et al. [17] created a hybrid AR model for simulating preoperative planning in metopic craniosynostosis surgery. Their team utilised patient imaging data to fabricate physical silicone head models replicating metopic craniosynostosis and overlaid AR-generated anatomical detail in real time to enrich spatial understanding of the deformity.

Preliminary results supported the utility of this mixed-reality platform as an effective adjunct to traditional surgical training. The authors noted that such tools could reduce planning errors, improve anatomical sensitivity, and shorten the learning curve for neurosurgical trainees prior to performing live procedures [17].

In addition, AR shows strong potential for remote surgical education and telementoring. Expert surgeons can use head-mounted mixed reality displays, such as the Microsoft HoloLens, to guide colleagues at distant medical centers through procedures in real time. These systems superimpose computer-generated 3D anatomical models and procedural cues onto the live surgical field, facilitating collaborative planning and intraoperative support. Cho et al. [18] validated the use of this technology on adult human skulls for collaborative craniofacial surgical planning. The AR interface provided superior 3D visualisation and depth perception of anatomical relationships compared to traditional 2D or conventional 3D display monitors. The system met established usability criteria and demonstrated the feasibility and effectiveness of holographic telementoring in enhancing remote surgical collaboration.

AR technology also holds significant potential as an educational tool to support patient and parental counselling [19]. By projecting virtual models onto a digital representation of the child’s head, AR can make complex anatomical concepts more accessible and engaging. In a study involving 412 caregivers of children with craniosynostosis, Chen et al. [19] compared preferences and understanding between 2D diagrams and patient-specific 3D-printed or AR models. The results showed that caregivers overwhelmingly preferred 3D-printed and AR models, finding them more effective for learning about craniosynostosis and skull anatomy. These models also helped reduce anxiety and build trust in the surgeon.

AR can improve caregiver understanding of what the surgery aims to correct, what to expect during recovery, and when to seek medical attention. Beyond education, AR can support post-operative rehabilitation by guiding head positioning and physical therapy exercises. It can visually demonstrate ideal post-surgical postures and identify high-pressure zones to avoid during the healing process, contributing to safer and more effective recovery.

### 3.4. Other Maxillofacial Uses

In the field of oral and maxillofacial surgery, AR is emerging as a transformative tool, particularly in complex procedures such as orthognathic surgery and tumour resection. AR technology enables the superimposition of virtual anatomical data directly onto the surgical field, enhancing precision and intraoperative decision-making.

For orthognathic surgery, AR systems can project preoperative virtual surgical planning data—such as osteotomy cutting lines and planned repositioning vectors—directly onto the patient’s maxilla and mandible [20,21]. This facilitates real-time surgical navigation and allows osteotomies to be executed with sub-millimetre accuracy. By aligning the surgical field with three-dimensional digital models, AR minimises the reliance on conventional guides and enhances the surgeon’s ability to execute complex bony movements precisely as planned.

In oncological surgery, AR can assist in the resection of lesions through delineating tumour margins and highlighting the spatial relationship to adjacent critical structures such as the inferior alveolar nerve, facial nerve branches, and major vessels [22,23]. This visual augmentation supports oncologically sound resections while preserving as much surrounding healthy tissue and function as possible, particularly vital in the head and neck region where functional and aesthetic outcomes are closely interlinked.

A key advantage of AR lies in its capacity to overcome the limitations of traditional 2D imaging. By providing surgeons with interactive, real-time 3D visualisations of the patient’s anatomy, AR allows for intuitive manipulation of views and dynamic exploration of anatomical planes. Surgeons can rotate, zoom, and interrogate structures virtually before and during the procedure, enhancing their spatial understanding of the surgical site [24,25,26].

**Figure 1:**
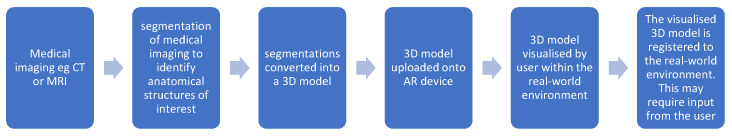
A conceptual figure illustrating the AR workflow from imaging through intraoperative use. In the case of intraoperative applications, the operating room is the real-world environment [30].

Head and neck reconstruction with fibula free-flap surgery involves making precise cuts to harvest the bone and prepare it for transfer to the recipient site. Traditionally, surgeons rely on physical cutting guides or freehand methods, but both approaches have limitations in adaptability and precision. During fibula free-flap harvesting, AR can display the preoperative 3D model of the fibula over the patient’s leg, helping to align the bone with the virtual template. This visualisation enables more accurate bone cuts, reducing the risk of errors such as over-cutting or misalignment, which can complicate the procedure and affect the functional outcome [27,28,29]. Furthermore, virtual segmentation of the fibula via medical imaging—CT or MRI—can be a useful training tool and has been shown to be acceptable to learners (Figure 1) [30].

### 3.5. Challenges and Limitations

Despite the promising applications of AR in craniofacial surgery, several challenges limit its widespread clinical adoption. Key technical hurdles include ensuring accurate and stable registration of virtual overlays onto patient anatomy, particularly in procedures involving soft tissue movement or long durations [1,31]. Ergonomic concerns also persist, as current AR headsets can be bulky, uncomfortable for extended use, and constrained by limited battery life [32].

Integration into existing surgical workflows poses another barrier. Many AR platforms require specialised setup or training, and seamless compatibility with hospital systems remains underdeveloped. Financial costs related to hardware, software, and personnel training further limit accessibility, particularly in resource-constrained settings [33].

Finally, while feasibility studies are encouraging, high-quality clinical data remain sparse. Most reports rely on small sample sizes and lack long-term outcome validation. Standardised protocols and cost-benefit analyses will be essential for scalable, evidence-based implementation of AR in routine craniofacial surgery.

### 3.6. Future Directions

#### Advancements in Eyewear and LiDAR

Advancements in eyewear technology are set to further embed AR into clinical workflows. Smart glasses equipped with touch-sensitive interfaces and voice command functionality have the potential to transform the way healthcare providers access and interact with patient information, enabling hands-free operation during crucial surgical procedures. Additionally, improvements in optical systems and sensor technology, particularly those leveraging LiDAR (Light Detection and Ranging), will enhance depth perception and environmental mapping, leading to more precise and dependable AR visualisations for surgical navigation [34].

## 4. Conclusions

This review is based solely on previously published literature; no new data were generated, nor original analyses performed.

Augmented reality (AR) represents a transformative tool in the surgical management of craniofacial deformities, particularly in the context of craniosynostosis. By enhancing preoperative planning, improving intraoperative precision, and offering novel opportunities for surgical education and remote collaboration, AR has the potential to significantly advance both patient outcomes and surgical efficiency. Feasibility studies and early clinical applications have demonstrated promising results in terms of accuracy, usability, and workflow integration.

The integration of AR into craniofacial surgery is already yielding measurable clinical benefits. AR has enabled enhanced surgical precision through accurate osteotomy placement and bone fragment alignment, particularly in cranial vault remodelling and midface reconstruction. Devices such as the HoloLens 2 have shown sub-millimetre accuracy in both preclinical and clinical settings, outperforming conventional navigation systems. Postoperative outcomes have also improved, with reductions in operative time and increased anatomical accuracy contributing to better functional and aesthetic results. For example, AR-assisted planning in synostotic plagiocephaly has been associated with significant reductions in intracranial volume asymmetry.

Beyond the operating room, AR is proving valuable in education and communication. Training platforms have improved spatial understanding and reduced planning errors among surgical trainees. In patient and caregiver engagement, AR models have enhanced comprehension and reduced anxiety, with over 90% of caregivers favouring 3D AR models over traditional 2D illustrations.

Nevertheless, key challenges remain, including technical limitations, ergonomic constraints, financial barriers, and the need for structured training. Continued innovation—especially in AI integration, robotic-assisted platforms, haptic feedback, and interoperability—will be critical to realising the full potential of AR in craniofacial surgery. Future research should prioritise clinical outcome validation, cost-effectiveness analyses, and the development of standardised protocols to support the safe, scalable, and evidence-based adoption of AR technologies.

To facilitate successful clinical integration, several actionable steps are recommended. These include initiating AR-assisted workflows in complex craniofacial procedures, standardising preoperative planning protocols, and developing institutional guidelines. Investment in surgical team training and IT infrastructure will be essential to support workflow integration. Early adoption should be encouraged through hybrid use with conventional navigation systems, accompanied by systematic tracking of outcomes such as operative time, accuracy, and recovery metrics. Finally, broader implementation will benefit from multicentre collaboration and rigorous cost-benefit analysis to ensure that AR technologies are accessible and effective across diverse healthcare environments, including resource-limited settings.

## Abbreviations

The following abbreviations are used in this manuscript:

AR	augmented reality
VSP	virtual surgical planning
VOSTARS	Video and Optical See-Through Augmented Reality Surgical System - CAD/CAM—Computer-Aided Design/Computer-Aided Manufacturing - AR-EM—Augmented Reality–Electromagnetic - PACS—Picture Archiving and Communication System - EHRs—Electronic Health Records - AI—Artificial Intelligence - LiDAR—Light Detection and Ranging
